# Quinia in Surgery and Obstetrics

**Published:** 1879-01-20

**Authors:** 


### *QUINIA IN SURGERY AND OBSTETRICS.*

The rapidly-extending use of quinia, and the excellent results that have followed its administration in many morbid conditions to which it was thought to be inapplicable, or rather in which its administration was not thought of at all in past times, must have attracted the attention of every reading member of the profession. To-day quinia stands out prominently as our greatest medicine, and malaria is more talked about and written about than any other source of disease.

Lately Dr. S. W. Gross, of Philadelphia, has recorded the success of quinia in his practice in preventing the untoward events which often follow surgical operations, and Dr. Hunter McGuire, of Richmond, in a recent number of the *Virginia Medical Monthly*, states it as his belief, founded on clinical experiment, that slight cinchonism prevents shock, or so lessens it as to make it inappreciable. It has been for some time the custom of Louisville surgeons to prepare their patients for operation by quinia, instead of by the ancient practice—sad to say, not yet wholly abandoned in certain quarters—of giving purgatives. They also give quinia after the operation, in order to ward off the evils which so often follow operations.

In obstetrical practice we have found quinia a most potent remedy. Indeed our experience is that puerperal fever, abscess of the breast, phlegmasia dolens, and the like, may be prevented with almost absolute certainty by the administration of quinia pricr and subsequently to childbirth. Iron is often a valuable ally of quinia, and should be used freely. The profession all over the world is rapidly awakening to the vast puissance of this *magnum bonum Dei*, and just in proportion as theory is discarded from our creed, and the results of clinical experiment are relied on as the source from which medical truth must come, shall we be led to adopt a sound, rational practice of physic? It is passing strange how theories in this enlightened, thinking, doubting age, still influence medical belief and practice. A theory has no more probable value than a lottery-ticket. By chance it may be correct, but it is immensely, incalculably more apt to be wrong. We cannot from our inner consciousness evolve a knowledge of the laws of nature. It is only by careful experiment that we may hope to discover the secrets of disease; and yet such reasoning as that of Prof. Lister leads astray not a few medical men. Prof. Lister says, in effect, that without the germ theory no rational explanation can be given of certain morbid processes; *ergo*, the germ theory must be true. This is not philosophical, this is not sound, this is not safe reasoning, and is not worthy the great though young science of medicine, which is now rapidly piling up sufficient facts to entitle it to be called a science.—*Louisville Medical News.*

